# Validity, Reliability, and Responsiveness of the Brief Pain Inventory in Inflammatory Bowel Disease

**DOI:** 10.1155/2016/5624261

**Published:** 2016-06-19

**Authors:** Lars-Petter Jelsness-Jørgensen, Bjørn Moum, Tore Grimstad, Jørgen Jahnsen, Randi Opheim, Ingrid Prytz Berset, Øistein Hovde, Roald Torp, Svein Oskar Frigstad, Gert Huppertz-Hauss, Tomm Bernklev

**Affiliations:** ^1^Health Science, Østfold University College, Postboks 700, 1757 Halden, Norway; ^2^Department of Gastroenterology, Østfold Hospital Trust, Fredrikstad, Norway; ^3^Department of Gastroenterology, Oslo University Hospital, Postboks 4959 Nydalen, 0424 Oslo, Norway; ^4^Institute of Clinical Medicine, University of Oslo, Postboks 1072 Blindern, 0316 Oslo, Norway; ^5^Department of Gastroenterology, Stavanger University Hospital, Armauer Hansensvei 20, 4011 Stavanger, Norway; ^6^Department of Gastroenterology, Akershus University Hospital, Postboks 1000, 1478 Lørenskog, Norway; ^7^Institute of Health and Society, University of Oslo, Postboks 1072 Blindern, 0316 Oslo, Norway; ^8^Department of Gastroenterology, Møre and Romsdal Hospital Trust, 6026 Ålesund, Norway; ^9^Department of Gastroenterology, Innlandet Hospital Trust, Kyrre Grepps Gate 11, 2819 Gjøvik, Norway; ^10^Department of Gastroenterology, Innlandet Hospital Trust, Postboks 104, 2381 Brumunddal, Norway; ^11^Department of Gastroenterology, Vestre Viken Hospital Trust, Postboks 800, 3004 Drammen, Norway; ^12^Department of Internal Medicine, Østfold Hospital Trust, Postboks 300, 1714 Grålum, Norway; ^13^Department of Gastroenterology, Telemark Hospital Trust, 3710 Skien, Norway; ^14^O&U, Telemark Hospital Trust, 3710 Skien, Norway

## Abstract

*Background and Aims*. No patient-reported outcome measures targeting pain have yet been validated for use in IBD patients. Consequently, the aim of this study was to test the psychometrical properties of the brief pain inventory (BPI) in an outpatient population with IBD.* Methods*. Participants were recruited from nine hospitals in the southeastern and western parts of Norway. Clinical and sociodemographic data were collected, and participants completed the BPI, as well as the Short-Form 36 (SF-36).* Results*. In total, 410 patients were included. The BPI displayed high correlations with the bodily pain dimension of the SF-36, as well as moderate correlations with disease activity indices. The BPI also displayed excellent internal consistency (Cronbach's alpha value of 0.91, regardless of diagnosis) and good to excellent test-retest values (intraclass correlation coefficient (ICC) 0.84–0.90 and Kappa values > .70). In UC, calculation of responsiveness revealed that only BPI interference in patients reporting improvement reached the threshold of 0.2. In CD, Cohen's *d* ranged from 0.26 to 0.68.* Conclusions*. The BPI may serve as an important supplement in patient-reported outcome measurement in IBD. There is need to confirm responsiveness in future studies. Moreover, responsiveness should ideally be investigated using changes in objective markers of inflammation.

## 1. Introduction

Inflammatory bowel diseases (IBDs), including ulcerative colitis (UC) and Crohn's disease (CD), are characterised by chronic, recurrent inflammation of the gastrointestinal (GI) tract [1, 2]. In UC, the inflammation is located in the colonic and rectal mucosa, whereas in CD, any part of the GI tract may be affected. Common IBD symptoms include diarrhoea, blood, and mucus in the stool, abdominal cramping, and fatigue [2–4].

In IBD, abdominal pain is also reported to be common and in most cases attributed to inflammation. Growing evidence suggests, however, that the aetiology of pain in IBD may be multifactorial [5]. It is well known that noninflammatory joint pain occurs frequently in IBD. Further, previous studies have shown that CD patients may develop immune-mediated extraintestinal manifestations, such as arthritis [6–8].

Pain is a subjective experience, and, therefore, the measurement of these symptoms is dependent on self-reports by patients. Several methods and questionnaires have been developed to measure pain, including visual analogue scales and numeric- and verbal-rating scales [9]. Developed by Cleeland and Ryan [10], the BPI was designed to measure (a) the subjective intensity of pain and (b) the impairment caused by pain. The BPI was originally developed in cancer treatment [10] and has been translated to several languages and validated as well [9, 11–14], but it has also been tested and used in nonmalignant conditions, such as osteoarthritis, rheumatoid arthritis, multiple sclerosis, diabetic neuropathy, and low back pain [15–19]. In addition to tools specifically developed to measure pain, pain is also an important part of several other patient-reported outcome measures (PROM), such as the generic health-related quality of life (HRQoL) questionnaire, Short-Form 36 (SF-36), and the disease-specific inflammatory bowel disease questionnaire (IBDQ) [20, 21].

Pain has been shown to have a causal influence on patient functioning [9]. Despite this, pain seems to be an underexplored area in IBD research, and, to our knowledge, no PROM specifically targeting pain have yet been validated for use in these patients.

Consequently, the aim of this study was to test the psychometrical properties of the brief pain inventory (BPI) in an outpatient population with IBD.

## 2. Materials and Methods

Participants were recruited from nine hospitals in the southeastern and western part of Norway as a part of a cross-sectional and longitudinal observational, multicentre study. Inclusion criteria were >17 years of age, a verified diagnosis of IBD (based on endoscopic, laboratory, and histological findings—Lennard-Jones criteria) [22], and the ability to read and write in Norwegian and to give written informed consent. Patients were excluded if the investigators found them to be unable to comply with the study procedures. The inclusion period was from March 2013 to April 2014. At each of the inclusion centres, a senior gastroenterologist was in charge of the study. The psychometrical testing of the BPI was performed using the quality recommendations of the COSMIN (Consensus-based Standards for the Selection of Health Measurement Instruments) checklist [23].

### 2.1. Sociodemographic and Clinical Data

Sociodemographic variables were self-reported by patients, which included age, gender, smoking habits, and self-perceived IBD symptoms. Self-perceived IBD symptoms were obtained through patient classification of IBD symptoms during the last 14 days. Four possible scores were used: no symptoms, mild symptoms (do not interfere with everyday activities), moderate symptoms (do interfere with everyday activities and may result in sick leave), and severe symptoms (unable to carry out everyday activities, on sick leave or hospitalised).

Disease activity was assessed through laboratory tests, faecal calprotectin (FeCal test—Calpro) and the activity indices Simple Clinical Colitis Activity Index (SCCAI) and Simplified Crohn's Disease Activity Index (SCDAI) [24, 25]. Phenotype was classified according to the Montreal classification. In addition, current use of medication was recorded from medical records.

### 2.2. Questionnaires

#### 2.2.1. The Brief Pain Inventory

The pain intensity section of the BPI consists of four items that are scored from 0 (no pain) to 10 (worst possible pain), whereas the functional interference section consists of seven items that are scored from 0 (no interference) to 10 (complete interference). A pain severity score is calculated from the mean of the four pain intensity items, and a pain interference score is calculated from the mean of the seven pain interference items [10]. In addition to the BPI intensity and interference items, the questionnaire also has four optional items that are not included in psychometrical testing according to the BPI user manual. The Norwegian translation of the BPI has been tested previously and Cronbach's alphas were 0.87 for the pain severity and 0.92 for the interference scales. Moreover, correlation between BPI pain severity and the European Organization for Research and Therapy of Cancer (EORTC) QLQ-C30 questionnaire item on pain intensity was 0.70 (*p* < 0.001). The correlation between BPI interference index and the EORTC QLQ-C30 item on pain influence on daily living was 0.62 (*p* < 0.001) [9].

#### 2.2.2. SF-36

The SF-36 is a well-validated, generic HRQoL questionnaire comprising 36 items [20, 26]. The 36 questions are divided into 8 multi-item scales, consisting of physical functioning (PF), role limitations because of physical problems (RP), bodily pain (BP), general health, vitality (VT), social functioning (SF), role limitations because of emotional problems (RE), and mental health (MH). For each question, the raw score was coded and transformed into a scale from 0 to 100, with 0 indicating the lowest level of function and 100 the highest level of function.

### 2.3. Statistical Analysis

To assess the characteristics of the sample, we used descriptive analysis, frequencies, and the *χ* ^2^ test.* Face validity* was tested by distributing the questionnaire to 15 patients before field testing, to receive their input on item content, scoring, and structure. The* Construct *of the BPI was tested by using a principal axis factoring, Oblimin rotation with Kaiser normalisation, and eigenvalues >1. To find the optimal cut-point for mild, moderate, and severe pain, we deployed previously described methods, using the average pain item of the BPI [27, 28]. Average pain was divided into 8 different schemes (using different options for upper values of mild and moderate pain). Multivariate analysis of variance was used, and the highest *F*-value (Wilks's lambda) was considered indicative of the scheme that was most useful for distinguishing mild, moderate, and severe pain.

*Concurrent validity* was tested through linear regression analysis, entering the well-established BP dimension of SF-36 as the dependent variable and average pain groups as the independent variable. We hypothesised that increased pain severity would be a negative predictor of BP.* Construct validity *was tested according to recommendations in the literature [29] using three approaches: (a) convergent validity, (b) discriminant validity, and (c) known-group validity.* Convergent validity *was calculated using binary correlation analysis (Spearman's rho) of the BPI, SF-36, and well-established disease activity indices. It was hypothesised that elevated pain (increased BPI scores) would correlate negatively with all SF-36 dimensions, but that the strongest negative correlation would be between the BPI and the bodily pain dimension. Increased disease activity indices were hypothesised to be positively associated with BPI scores. In UC, none of the items in the SCCAI specifically measure pain. However, we chose to correlate the BPI against the SCCAI items (a) general condition and (b) complications (e.g., including joint pain). In CD, the BPI was correlated with the SCDAI items (a) abdominal pain and (b) complications.* Discriminant validity* was calculated by comparing the correlation between the BPI items and their hypothesised dimension, with its correlation to other dimensions.* Known-group validity* was tested through one-way analysis of variance, by comparing mean BPI scores in patients reporting no, mild, moderate, or serious IBD symptoms. Moreover, we hypothesised that the SF-36 bodily pain would be negatively associated with increased pain level (BPI average pain). Post hoc Scheffe test was used to control for multiple comparisons.* Floor and ceiling* effects were investigated by calculating the percentage of patients scoring either the lowest or highest possible score in individual items, as well as in dimensional scores. If the number of lowest or highest possible scores on the BPI exceeded 15%, this was, according to recommendations [30], regarded as indicative of floor or ceiling effects.* Internal consistency reliability* was tested with Cronbach's alpha. When answering the retest, patients were asked to indicate whether their condition was unchanged, deteriorated or improved since baseline.* Test-retest reliability* was measured using the intraclass correlation coefficient (ICC, two-way mixed, single measure). Patients self-reported their perceived disease state at the second time of BPI assessment (4–6 weeks apart) using a question with three potential answers: “Compared to last time you completed the questionnaire, how do you evaluate your IBD condition today? (A) Unchanged (B) Improved, or (C) Deteriorated.” Based on this item, ICC values were calculated among those patients reporting to be in a stable condition.* Responsiveness* was calculated by comparing the BPI scores on baseline to those after 4–6 weeks in patients who reported either worsening or improvement in IBD symptoms. Both Guyatt's statistics and Cohen's *d* were used to calculate responsiveness. Guyatt's statistics was performed by dividing the mean change in individuals reporting either improvement or deterioration of symptoms with the standard deviation of the change score in those unchanged. Cohen's *d* effect size was calculated by comparing the mean difference between groups, divided by the pooled standard deviation. Operational definitions of 0.2, 0.5, and 0.8 were categorised as small, medium, and large, respectively. Missing data were treated as recommended in the literature; if data in half or less than half of the items within a scale were missing, they were replaced by the mean value of the respondent's completed items in the same scale [31]. All tests were two-sided, with a 5% significance level, and were performed by the use of Predictive Analytics Software, PASW, version 23.0 (SPSS Inc., 233 S. Wacker Drive, Chicago, Illinois, United States).

### 2.4. Ethical Considerations

Participation in the study was based on written informed consent and performed in accordance with the principles of the revised Helsinki Declaration. Approval was obtained from the Regional Ethics Committee (reference number: 2012/845/REK Sør-Øst A).

## 3. Results

In total, 452 patients were eligible and were invited to participate in the study. Further, 414 patients (91.6%) gave written informed consent, while 4 of these patients were excluded because the number of missing data exceeded 50%, leaving the number included for analyses at 410. Of these, 230 were diagnosed with CD and 180 with UC. Baseline characteristics of the included patients are presented in Table 1. No significant differences were found in gender or age between patients declining participation, being excluded because of missing values or those included in analyses. Data on the diagnosis of those declining participation, however, were not available. In 325/410 patients, calprotectin was available. A significant increase in calprotectin (*p* = 0.001) levels according to patient-perceived IBD symptoms was observed (no symptoms mean = 184; mild symptoms mean = 203; moderate symptoms mean = 222; and severe symptoms mean = 440).

After inviting all 410 patients from baseline to complete the BPI a second time, 243 responded, corresponding to 59% of the original sample (CD 130/230; UC 113/180). None of those 242 patients at the retest had missing values on the BPI. In CD, 110 patients reported that their condition was unchanged compared with baseline, whereas 14 reported symptom improvements and 5 deterioration. The comparable numbers in UC were 86 unchanged, 20 improved, and 8 worsened.

Overall BPI scores according to diagnosis are presented in Table 2. In UC, floor effects in individual items varied from 25% (pain average) to 75.6% (pain interference, walking). In the BPI intensity dimension, the floor effect was 22.8%, whereas the comparable number in the BPI interference dimension was 33.9%. In CD, floor effects in individual items varied from 23% (pain average) to 75.7% (pain interference, walking). In the BPI dimensional scores, the floor effect was 21.3% for intensity and 35.2% for interference. Ceiling effects did not exceed 15% in either UC or CD.

Evaluating cut-off values for mild, moderate, and severe pain resulted in the optimal cut-off being (a) a score of 0 = no pain; (b) 1–3 = mild pain; (c) 4–6 = moderate pain; and (d) ≥7 = severe pain (*F*-value = 11.8). Using this classification, the distribution among patients was no pain (CD, *n* = 53; UC, *n* = 45), mild pain (CD, *n* = 129; UC, *n* = 92), moderate pain (CD, *n* = 33; UC, *n* = 24), and severe pain (CD, *n* = 15; UC, *n* = 19).

Analysis of construct, omitting values below 0.40, revealed a two-factor solution explaining 68% of the variance. The factor solution was equal when analysed separately for UC and CD. Items with high loadings in factor one (0.73–0.89) included all four original BPI intensity items (pain worst, least, average, and now), whereas items with high loadings in factor two (0.58–0.93) included the seven original BPI interference items (general activity, mood, walking, work, relationship, sleep, and enjoyment).

### 3.1. Validity

Testing of* face validity* revealed no problematic issues regarding either item content or scoring. However, two patients called to attention the first and optional BPI screening item: “Throughout our lives, most of us have had pain from time to time (such as minor headaches, sprains, and toothaches). Have you had pain other than these everyday kinds of pain today?” This might potentially, if answered no, cause respondents to leave the rest of the items unanswered.

Calculation of concurrent validity revealed that increased pain levels were predictive of more BP on the SF-36 (CD: *β* = −.63, *p* < 0.001, UC: *β* = −.65, *p* < 0.001). Construct validity tests revealed moderate to high negative correlation between the BPI intensity and interference dimensions and the bodily pain dimension of the SF-36. In addition, the BPI dimensions were positively correlated to the SCCAI and SCDAI (Table 3). Estimation of discriminant validity showed the highest correlation between items and their hypothesised dimension (Table 4), as well as lower correlation coefficients on SF-36 dimensions measuring other aspects than pain (Table 3).

**Figure 1:**
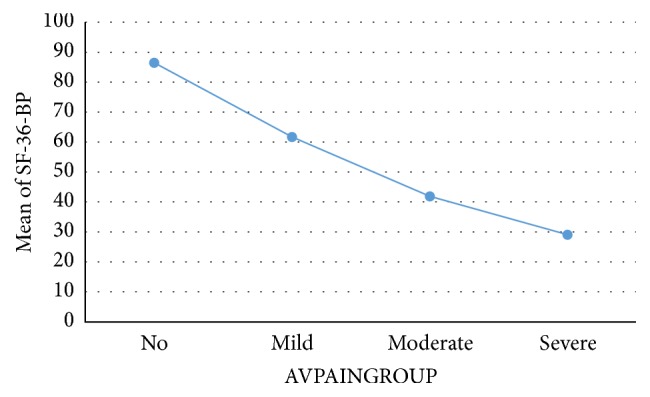
Mean SF-36 bodily pain (BP) in ulcerative colitis according to average pain severity. SF-36: short-Form 36. AVPAINGROUP: average pain grouped according to cut-off values 0 = no, 1–3 mild, 4–6 moderate, and ≥7 severe. Mean bodily pain scored from 0 to 100. A higher score indicates less problems.

**Figure 2:**
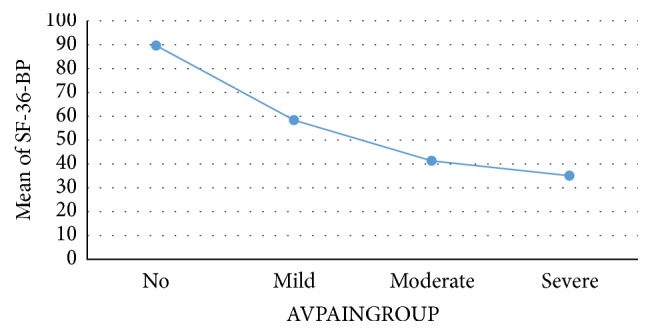
Mean SF-36 bodily pain (BP) in Crohn's disease according to average pain severity. SF-36: Short-Form 36. AVPAINGROUP: average pain grouped according to cut-off values 0 = no, 1–3 mild, 4–6 moderate, and ≥7 severe. Mean bodily pain scored from 0 to 100. A higher score indicates less problems.

Known-group validation showed a tendency of elevated BPI levels as the patients' subjective IBD symptoms increased (Table 5). In UC, post hoc Scheffe test revealed significant BPI differences in the following symptom groups: no-moderate (*p* < 0.05); no-serious (*p* < 0.01); mild-serious (*p* < 0.01); and moderate-serious (*p* < 0.01). In CD, significant effects were seen in no-moderate/serious (*p* < 0.01); mild-moderate (*p* < 0.01); and mild-serious (*p* < 0.05). Moreover, as presented in Figures 1 and 2, the BP dimension of the SF-36 decreased according to pain severity.

### 3.2. Reliability and Responsiveness

Cronbach's alpha calculated, for UC and CD separately, revealed excellent alpha values regardless of diagnosis (alpha 0.91). Test-retest reliability of the BPI is presented in Table 2. In individual and dimensional BPI scores, the strength of agreement between the two occasions (ICC) was high (see Table 2 for details). The Spearman's correlation of BPI intensity scores from baseline to follow-up was .86 and .85 in CD and UC, respectively. The corresponding figure for BPI interference was .90, regardless of diagnosis. When comparing pain categories no, mild, moderate, and severe, Kappa values were .70 in CD and .72 in UC.

In patients reporting that their symptoms were either improved or deteriorated since baseline assessment, BPI values tended to be decreased or increased, respectively (Table 5). A Guyatt's statistics greater than 1.00 was regarded as indicative of high responsiveness, whereas scores greater than 0.20 were considered adequate. In UC, all scores, except BPI interference in patients reporting improvement, were lower than 0.20. In CD, all scores exceeded 0.20, and, in patients reporting improvement, the BPI interference scores were greater than 1.00, whereas, in patients reporting deterioration, the BPI intensity scores were greater than 1.00. Cohen's *d* effect sizes were generally lower in UC than in CD. In CD, all scores, except BPI interference in patients who reported a worsening, were moderate (Table 6). In UC, only BPI interference in patients who reported improvement reached the cut-off of small effect size.

## 4. Discussion

This study revealed that the BPI is a valid and reliable tool for assessment of pain intensity and interference in both UC and CD. These findings are consistent with studies in other autoimmune diseases such as multiple sclerosis, rheumatoid arthritis, and diabetes [16, 18, 19]. Based on findings from the current study, the BPI consequently adds to the existing body of PROMs in IBD and may become an important supplement when pain measurement is needed.

Validation is a process involving several stages, all aiming to determine whether an instrument measures what it is intended to measure or is useful for its intended purpose [29]. Face validity testing in a subgroup of patients revealed no problematic issues regarding item content or scoring. However, some remarks were raised concerning the optional first item of the BPI, which could lead to a misunderstanding of whether or not patients should fill out the remaining questionnaire. The completeness of the BPI was, however, high, with merely four patients being excluded from analyses because of missing data. Consequently, we conclude that the level of misunderstanding was low in the current study. On the contrary, based on patient input and to avoid misunderstanding of scoring and interpretation, we suggest that it might be better to place the mandatory items of the BPI first, followed by optional items.

Other than a validation study in cancer patients [9], factor analyses of the BPI in several other languages have identified a two-factor model with pain intensity items and interference items loading on the two factors. These studies and ours have used similar statistical methods, including principal axis factoring and Oblimin rotation. The rationale for using Oblimin rotation was based on the assumption that BPI items were not orthogonal or correlated [29]. Our analysis yielded a two-factor solution that was consistent with the original structure and validation of the BPI [10].

We observed a marked floor effect in BPI score regardless of diagnosis, which implies that the number of patients scoring the lowest possible score exceeded 15% [30]. A potential explanation for this finding is that pain measurement is based on measuring the existence of a specific problem or not (pain versus no pain) compared with generic HRQoL instruments, for example, [29]. Consequently, despite a high number of participants reporting no problems in the BPI items, this does not necessarily indicate a limitation of the BPI. Indeed, for descriptive and evaluative purposes, the assessment of pain must be able to detect the number of patients with a low versus high symptom burden [29]. A manifest floor effect has also been observed elsewhere [9]. However, no ceiling effects were observed in our study, and this may indicate, compared with cancer patients, for example, that few IBD patients experience the highest possible pain burden [9]. Obviously, this cannot be generalised beyond our outpatient population.

Out of the eight SF-36 dimensions, the BPI displayed the highest correlations with the bodily pain dimension. Moreover, weak to modest correlations were found related to aspects of disease activity indices. Because joint pain and rheumatic disorders are well-known extraintestinal manifestations of IBD [8, 32, 33], we chose to correlate the BPI against the complication item of the SCCAI and SCDAI, which is because these items capture some of these symptoms. Stomach pain, however, is only captured by the SCDAI and not the SCCAI, which of course may limit the exact interpretability in UC.

Optimally, a PROM should be able to discriminate between groups of patients anticipated to have differences in health states. Our results indicate that the BPI can capture these differences. Moreover, when categorising patients into no, mild, moderate, and severe pain, the SF-36 BP dimension dropped accordingly.

The sample size needed in test-retest analysis has been the subject of some debate. Some have advocated that a sample size of 50 could be sufficient or a starting point [34]. Others have highlighted the need for larger sample sizes and more robust test-retest data [35]. In the current study, a convenient sample of 242 patients was included in test-retest, of which 196 patients reported an unchanged condition. Therefore, the test-retest analysis in the current study is based on a robust sample of patients. Our results showed that, within a timeframe of 4 to 6 weeks, the BPI displayed good ICC values in individual items and dimensional scores. Moreover, following recommendations [35], test-retest analysis was performed by the same assessor as at baseline. Both assessor and subjects were blinded to performance and scores from baseline during the retest of the BPI.

A central aspect of a PROM measure is the ability to respond to relevant changes in a particular condition, also known as responsiveness. The number of patients reporting either deterioration or improvement in this study was generally low. Although there was a tendency of scores corresponding with change in IBD condition, these findings should be interpreted with caution. Both Guyatt's statistics and Cohen's *d* effect sizes revealed that the responsiveness was higher in CD than in UC. In UC, the responsiveness may be questioned because only one of the BPI dimensions reached a Guyatt's statistics and Cohen's *d* above the threshold of 0.2. There may of course be several explanations to this finding, including inherent differences between UC and CD, as well as lack of precision in the question used to capture change from baseline to retest.

The study has some limitations. Because we recruited only hospital outpatients, our sample may not be representative of a community sample of IBD patients. Consequently, we cannot conclude about the BPI's psychometrical properties in IBD at large. Further, we evaluated change using merely the patient's subjective experience of having an unchanged, improved, or worse IBD condition at time of retest. Ideally, because subjective evaluation of health status may increase the risk of bias, we should have evaluated change using an objective marker of inflammation, such as calprotectin. In addition, we were not able to calculate minimal important difference (MID), which could have been useful in determining whether the observed change is meaningful to patients or not. MID is limited to the subgroup of people who are deemed to have had minimal change, but we did not include an adequate anchor to calculate these values.

In conclusion, this study is the first to demonstrate that the BPI is an easily scored, valid, reliable measure of pain in IBD patients. Consequently, the BPI may serve as an important supplement in patient-reported outcome measurement in IBD. Although indications of responsiveness were found, there is still a need to confirm these in future studies. Moreover, responsiveness should ideally be investigated using changes in objective markers of inflammation, such as calprotectin.

## Supplementary Material

The Brief Pain Inventory is provided as supplementary material.

## Figures and Tables

**Table 1:** Sociodemographic and clinical data according to diagnosis.

	UC (*n* = 180)	CD (*n* = 230)	*p* value
Age mean (SD)	40.8 (12.6)	40.7 (13.0)	ns
Age (range)	18–76	18–77	ns
Gender			
Female	87	114	
Male	93	116	ns
Time since diagnosis (years)	8.8 (8.2)	13.6 (10.5)	<0.001
SCCAI total score	3.4 (3.1)		
SCDAI total score		4.7 (3.8)	
UC extent^¶^			
E1—proctitis	20 (11.1%)		
E2—left-sided colitis	58 (32.2%)		
E3—extensive colitis	102 (56.7%)		
CD localization^¶^			
L1—terminal ileum		75 (32.6%)	
L2—colon		47 (20.4%)	
L3—ileocolon		76 (33.0%)	
L4—upper GI		32 (13.9%)	
CD upper GI-modification			
L1 + L4		8 (25.0%)	
L2 + L4		6 (18.8%)	
L3 + L4		18 (56.3%)	
CD behavior^¶^			
B1—nonpenetrating/nonstricturing		117 (50.9%)	
B2—penetrating		30 (13.0%)	
B3—stricturing		83 (36.1%)	

UC: ulcerative colitis, CD: Crohn's disease, SD: standard deviation, SCCAI: Simple Clinical Colitis Activity Index, SCDAI: Simplified Crohn's Disease Activity Index, ^¶^Montreal classification, ns: nonsignificant. Figures are in mean and standard deviation if not otherwise noted.

**Table 2:** Overall mean (SD) BPI scores according to diagnosis and test-retest scores in patients with an unchanged condition.

BPI item	Ulcerative colitis				Crohn's disease			
	Overall T1 (*n* = 180)	Unchanged T1 (*n* = 86)	Unchanged T2 (*n* = 86)	ICC^¶^	Overall T1 (*n* = 230)	Unchanged T1 (*n* = 110)	Unchanged T2 (*n* = 110)	ICC^¶^
Pain worst	2.7 (2.4)	2.4 (2.4)	2.2 (2.3)	0.85	2.5 (2.3)	2.7 (2.1)	2.9 (2.4)	0.78
Pain least	1.2 (1.6)	0.9 (1.5)	1.0 (1.7)	0.81	1.0 (1.3)	0.9 (1.3)	1.0 (1.3)	0.73
Pain average	2.8 (2.3)	2.3 (2.2)	2.2 (2.1)	0.83	2.6 (2.3)	2.6 (2.2)	2.7 (2.2)	0.79
Pain now	1.5 (1.9)	1.2 (1.7)	1.4 (1.7)	0.86	1.4 (1.7)	1.5 (1.7)	1.7 (2.0)	0.85
General activity	2.4 (2.5)	2.0 (2.3)	2.0 (2.3)	0.92	2.4 (2.8)	2.3 (2.6)	2.4 (2.5)	0.90
Mood	2.4 (2.6)	2.0 (2.2)	2.0 (2.2)	0.92	2.4 (2.6)	2.6 (2.5)	2.5 (2.6)	0.89
Walking	0.8 (1.8)	0.7 (1.5)	0.6 (1.2)	0.83	0.8 (1.7)	0.8 (1.7)	0.7 (1.6)	0.84
Work	2.1 (2.6)	1.7 (2.5)	1.6 (2.3)	0.87	2.2 (2.8)	2.2 (2.8)	2.2 (2.8)	0.87
Relationship with others	2.0 (2.5)	1.7 (2.5)	1.5 (2.1)	0.85	2.0 (2.5)	2.1 (2.6)	2.1 (2.6)	0.90
Sleep	1.9 (2.7)	1.9 (2.7)	1.7 (2.4)	0.90	1.8 (2.5)	1.8 (2.5)	1.9 (2.5)	0.84
Enjoy	2.4 (2.7)	2.1 (2.5)	1.9 (2.2)	0.82	2.3 (2.7)	2.3 (2.6)	2.3 (2.7)	0.91
BPI intensity^±^	8.2 (7.5)	6.9 (7.0)	6.8 (6.9)	0.89	7.5 (6.4)	7.7 (6.2)	8.3 (6.9)	0.84
BPI interference^±^	14.0 (15.2)	12.2 (13.8)	11.4 (12.2)	0.89	13.9 (15.4)	14.2 (15.6)	14.1 (14.7)	0.90

SD: standard deviation, BPI: brief pain inventory, T1: baseline, T2: retest (4–6 weeks after baseline measurement), ICC: intraclass correlation coefficient, ^¶^between patients with unchanged condition from T1 to T2, ^±^sum scores of individual items.

**Table 3:** Spearman correlation between the BPI dimensions, the dimensions of the short-form 36 (SF-36), and disease activity indices.

	Ulcerative colitis		Crohn's disease	
	BPI intensity	BPI interference	BPI intensity	BPI interference
*SF-36 dimensions*				
PF	−.50	−.60	−.44	−.44
RP	−.47	−.53	−.37	−.47
BP	**−.70**	**−.73**	**−.76**	**−.73**
GH	−.49	−.54	−.36	−.40
VT	−.45	−.49	−.30	−.32
SF	−.42	−.49	−.33	−.43
RE	−.35	−.36	−.23	−.32
MH	−.40	−.41	−.26	−.32
*Disease activity indices*				
SCCAI general condition	**.42**	**.53**		
SCCAI complications	.32	.31		
SCDAI abdominal pain			**.51**	**.48**
SCDAI complications			.29	.28

BPI: brief pain inventory, PF: physical functioning, RP: role physical, BP: bodily pain, GH: general health, VT: vitality, SF: social functioning, RE: role emotional, MH: mental health, SCCAI: Simple Clinical Colitis Activity Index, SCDAI: Simplified Crohn's Disease Activity Index. Highest correlation coefficients are in bold face.

**Table 4:** Spearman correlation between individual BPI items and BPI dimensions.

BPI item	Ulcerative colitis (*n* = 180)		Crohn's disease (*n* = 230)	
	BPI intensity dimension	BPI interference dimension	BPI intensity dimension	BPI interference dimension
Pain worst	**.93**	.86	**.92**	.84
Pain least	**.85**	.69	**.77**	.63
Pain average	**.92**	.77	**.88**	.70
Pain now	**.90**	.79	**.86**	.74
General activity	.83	**.95**	.82	**.94**
Mood	.83	**.93**	.78	**.94**
Walking	.53	**.67**	.52	**.61**
Work	.75	**.91**	.74	**.91**
Relationship with others	.77	**.89**	.73	**.88**
Sleep	.71	**.81**	.70	**.83**
Enjoy	.79	**.94**	.75	**.90**

BPI: brief pain inventory. Highest correlation is in bold face.

**Table 5:** Known group validation of BPI scores according to self-assessed symptom burden at baseline.

	Ulcerative colitis (*n* = 180)	
	BPI intensity	BPI interference
*IBD symptoms*		
No (*n* = 31)	3.5 (5.3)	3.9 (6.8)
Mild (*n* = 65)	7.6 (6.5)	12.6 (14.4)
Moderate (*n* = 61)	8.5 (7.2)	15.5 (15.4)
Severe (*n* = 23)	15.3 (8.5)	27.9 (14.7)

	Crohn's disease (*n* = 230)	
	BPI intensity	BPI interference

*IBD symptoms*		
No (*n* = 38)	3.7 (4.9)	4.4 (7.9)
Mild (*n* = 79)	6.2 (5.5)	9.9 (13.2)
Moderate (*n* = 83)	9.7 (6.4)	18.9 (15.8)
Severe (*n* = 30)	10.0 (7.3)	22.3 (17.5)

BPI: brief pain inventory, IBD: inflammatory bowel disease.

**Table 6:** BPI sensitivity to change among patients reporting improved or worse symptoms from baseline.

	Ulcerative colitis						Crohn's disease					
	Improved			Worse			Improved			Worse		
	T1 (*n* = 20)	T2 (*n* = 20)	Cohen's *d*	T1 (*n* = 8)	T2 (*n* = 8)	Cohen's *d*	T1 (*n* = 14)	T2 (*n* = 14)	Cohen's *d*	T1 (*n* = 5)	T2 (*n* = 5)	Cohen's *d*
BPI intensity	10.0 (7.7)	9.2 (6.5)	0.11	9.4 (10.4)	10.4 (8.6)	−0.10	12.9 (6.5)	8.8 (6.3)	0.64	10.5 (5.9)	14.5 (5.7)	−0.68
BPI interference	18.5 (20.2)	13.6 (16.6)	0.26	16.0 (16.6)	16.4 (18.5)	−0.02	27.9 (20.5)	15.8 (17.1)	0.64	17.5 (16.5)	22.3 (19.9)	−0.26

BPI: brief pain inventory, T1: baseline, T2: retest (4–6 weeks after baseline). Figures in mean and standard deviation. Cohen's *d* effect size: 0.2 small, 0.5 moderate, and 0.8 large.
